# ALK‐rearranged squamous cell carcinoma of the lung

**DOI:** 10.1111/1759-7714.13818

**Published:** 2021-02-09

**Authors:** Qiyi Meng, Yujie Dong, Hong Tao, Liang Shi, Li Tong, Junfang Tang, Shucai Zhang, Zhe Liu

**Affiliations:** ^1^ Department of Medical Oncology, Beijing Chest Hospital Capital Medical University, Beijing Tuberculosis and Thoracic Tumor Research Institute Beijing China; ^2^ Department of Pathology, Beijing Chest Hospital Capital Medical University, Beijing Tuberculosis and Thoracic Tumor Research Institute Beijing China

**Keywords:** ALK inhibitor drug, ALK rearrangement, immunotherapy, PDL1, squamous cell carcinoma

## Abstract

**Background:**

ALK rearrangement is a very rare subset of squamous cell carcinoma (SCC) and one of the clinical features in patients is lack of data. Here, we report eight patients diagnosed with SCC of the lung harboring ALK rearrangement.

**Methods:**

We collected primary NSCLC samples at the Beijing Chest Hospital between January 2012 and December 2018 for Ventana (D5F3) immunohistochemical detection. Among the 148 patients was diagnosed ALK‐rearranged non small cell lung cancer (NSCLC), only eight cases was SCC. We collected patients information from electronic patent records (EPRs).

**Results:**

The eight cases of SCC were diagnosed by immunohistochemistry (IHC). Two were given crizotinib as second‐line therapy. One patient had stable disease (SD) and progression‐free survival (PFS) of six months. The other patient had progressive disease (PD) but PFS was only one month. The side effects were tolerable. This report identified 31 cases of ALK rearrangement in SCC patients from a literature search (including the eight patients in this study). These fusion genes are often seen in a younger age group (mean age: 55.6 years) and non‐smokers (18/31, 58.1%). A total of 20 cases received an ALK inhibitor as first‐ or second‐line treatment which included 11 with a partial response (PR), four with SD, and five with PD. The DCR and ORR was 75.0% (15/20) and 55.0% (11/20), respectively. The median duration time of therapy was 6.4 ± 4.4 months.

**Conclusions:**

Patients with ALK‐rearranged SCC obtained clinical benefit from ALK‐inhibitor therapy, especially those who were non‐smokers and whose tumors had been identified by IHC+/FISH+.

## Graphical abstract

Clinical benefit was obtained from ALK‐inhibitor treatment in ALK‐rearranged SCC especially in non‐smokers and patients whose tumors had been identified by IHC+/FISH+.

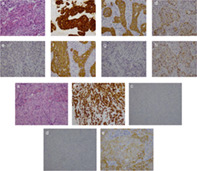

## INTRODUCTION

Lung cancer has among the highest morbidity and mortality of all cancer types, and is responsible for the highest rate of cancer‐related mortality worldwide.^1^ Primary lung cancer is mainly divided into two pathological types: small cell lung cancer (SCLC) and non‐small cell lung cancer (NSCLC), which mainly include adenocarcinoma (ADC), squamous cell cancer (SCC) and other subtypes.^2^ Treatment methods for lung cancer mainly include surgical resection, chemotherapy and molecular targeted therapy.^3^

Numerous oncogenic driver mutations have been identified in patients with NSCLC. Echinoderm microtubule‐associated protein‐like 4 and anaplastic lymphoma kinase rearrangement fusion genes (EML4‐ALK) have been previously identified in approximately 5% of NSCLC patients, a population that consists mostly of adenocarcinoma patients.^4^ These fusion genes are often seen in a younger age group of never or light ex‐smokers. Patients with SCC who harbor the ALK rearrangement are extremely rare (only 1%).^5^

Targeted treatment of metastatic ALK‐rearranged non‐small cell lung cancer with ALK inhibitors leads to higher response rates and improves progression‐free survival (PFS) relative to conventional chemotherapy regimens.^6^

However, only limited data on ALK rearrangement in SCC are available and we seldom have the opportunity to see those patients in daily clinical practice. Here, in this study, we report eight cases with ALK‐positive SCC together with a review of the literature.

## METHODS

### Clinical data collection

We collected primary NSCLC samples at the Beijing Chest Hospital between January 2012 and December 2018 for Ventana (D5F3) immunohistochemical detection. Among the 148 patients with pathologically diagnosed ALK‐rearranged NSCLC, overexpression of ALK protein occurred in eight patients with SCC, as confirmed by immunohistochemistry (IHC).

### Treatment response evaluation and follow‐up

Patient treatment information was obtained from electronic patient records (EPRs). The tumor response in patients who received standard chemotherapy was assessed every two cycles. In the patients who received an ALK inhibitor, the tumor response in this group was assessed after the first cycle (every 28‐day cycle) of treatment and subsequently after every two cycles. Tumor responses were assessed using the Response Evaluation Criteria in Solid Tumors (RECIST) version 1.1^7^ During follow‐up, CT scans of the thorax and enhanced MRI of the brain were used to assess the disease. Responses to treatment were reported as complete response (CR), partial response (PR), stable disease (SD), or progressive disease (PD).

### Regular IHC and Ventana IHC analysis

#### Immunohistochemistry analysis

There were five surgically resected samples and eight biopsy samples assessed by immunohistochemistry analysis including P40, P63, cytokeratin 5/6, CK7, thyroid transcription factor 1(TTF‐1), and napsin‐A. IHC revealed that the tumor cells showed diffuse staining and were positive for P40, P63, CK5/6, CK7, but were completely negative for thyroid transcription factor‐1 (TTF‐1) and napsin‐A. Although few cells (less than 5% of all) showed adenomatous differentiation, most had typical characteristics of SCC. Based on these findings, the results confirmed the histological type was SCC.

#### ALK immunohistochemistry analysis

Sections of formalin‐fixed and paraffin‐embedded (FFPE) tissue 4 μm thick were stained with the Ventana ALK (D5F3) rabbit monoclonal primary antibody, together with rabbit monoclonal negative control immunoglobulin, Optiview DAB IHC detection kit, and an Optiview amplification kit on a Ventana BenchMark ULTRA stainer (Ventana Medical Systems). This procedure was performed according to the manufacturer's instructions.^8^, ^9^

Immunoreactivity was evaluated as positive when the tumor (any percentage of positive tumor cells) showed intense granular cytoplasmic staining. The test results were evaluated using light microscopy. A binary method of interpretation was used as follows: strong granular cytoplasmic staining (any percentage) in the tumor cells was scored EML4‐ALK (+); otherwise, and they were scored EML4‐ALK (−).

### Statistical analysis

The association between ALK rearrangement status and clinicopathological data was assessed using SPSS software, version 17.0 (SPSS, Chicago, IL, USA), and survival analysis was performed using Fisher's exact test and the Kaplan–Meier test. A two‐sided *p*‐value < 0.05 was considered statistically significant.

## RESULTS

### Clinicopathological features of ALK‐rearranged SCC (TABLE 1)

Two of the eight cases in this study were female and six were male. Five of eight patients with ALK‐rearranged SCC were ex‐ or current smokers. Pathological staging was I–IV and included one patient (stage I), one patient (stage II), three patients (stage III) and three patients (stage IV), respectively.

**TABLE 1:** Clinical details of patients

	Age (y)	Sex	Method of diagnosis and/type of tissue sample	ALK detection	Stage	Smoking history pack‐year	Prior treatment	ALK inhibitor	Efficacy	PFS (m)
Case 1	54	F	Bronchial biopsy	IHC	IV	Non‐smoker	PDC	Crizotinib	SD	6.0
Case 2	76	M	Lung biopsy	IHC	IV	Smoker	PDC	None		
Case 3	53	M	Lung biopsy operation	IHC,	IIIa	Smoker	Adjunctive PDC	None		
Case 4	35	F	Lung biopsy cervical lymph node	IHC,	IV	Non‐smoker	PDC	Crizotinib	PD	1.0(Dead)
Case 5	64	M	Bronchial biopsy/surgery	IHC,	II	Smoker	None	None		
Case 6	64	M	Bronchial biopsy/surgery	IHC	IIIa	Smoker	Adjunctive PDC	None		
Case 7	63	M	Bronchial biopsy/surgery	IHC	I	Non‐smoker	PDC	None		
Case 8	64	M	Bronchial biopsy /surgery	IHC	IIIa	Smoker	Adjunctive PDC	None		

Abbreviations: F, female; FISH, fluorescence in situ hybridization; M, male; m, months; PD, progressive; PDC, platinum‐doublet chemotherapy; PFS, progression‐free survival; PR, partial response; RT‐PCR, reverse transcription polymerase chain reaction; SD, stable disease; y, year.

SCC was confirmed by typical histopathological features. All eight cases were positive for CK5/6, CK7, p63 and/or p40 expression, negative for TTF‐1 and napsin A. Ventana IHC (D5F3) confirmed the presence of ALK in all cases.

Five patients received surgery. Three of five cases (Cases 3, 6 and 8) received adjunctive chemotherapy and no recurrence was observed after regular follow‐up. Intrapulmonary metastasis recurred 14 months after surgery in Case 5 and the patient abandoned further treatment. There was a recurrence in Case 7 with local metastasis 25 months after surgery who subsequently received radiotherapy. Then, 10 months later, the patient suffered from intrapulmonary and bone metastasis and received first‐line chemotherapy with a combination of four cycles of gemcitabine and carboplatin to which there was no treatment response. The patient refused a further course of treatment for economic reasons.

Three patients had advanced NSCLC. Case 2 was a male aged 76 years. He was unable to afford the high cost of targeted therapy, and only accepted one cycle of chemotherapy before refusing subsequent treatment. The other two patients with ALK‐rearranged SCC had previously undergone standard chemotherapy. Two patients were treated with crizotinib and then switched regimens when they were diagnosed with PD during chemotherapy. One received a SD response; unfortunately, the second patient underwent crizotinib for only one month's duration and then died as a result of cancer progression.

#### Case 1: An ALK inhibitor benefit

**FIGURE 1:**
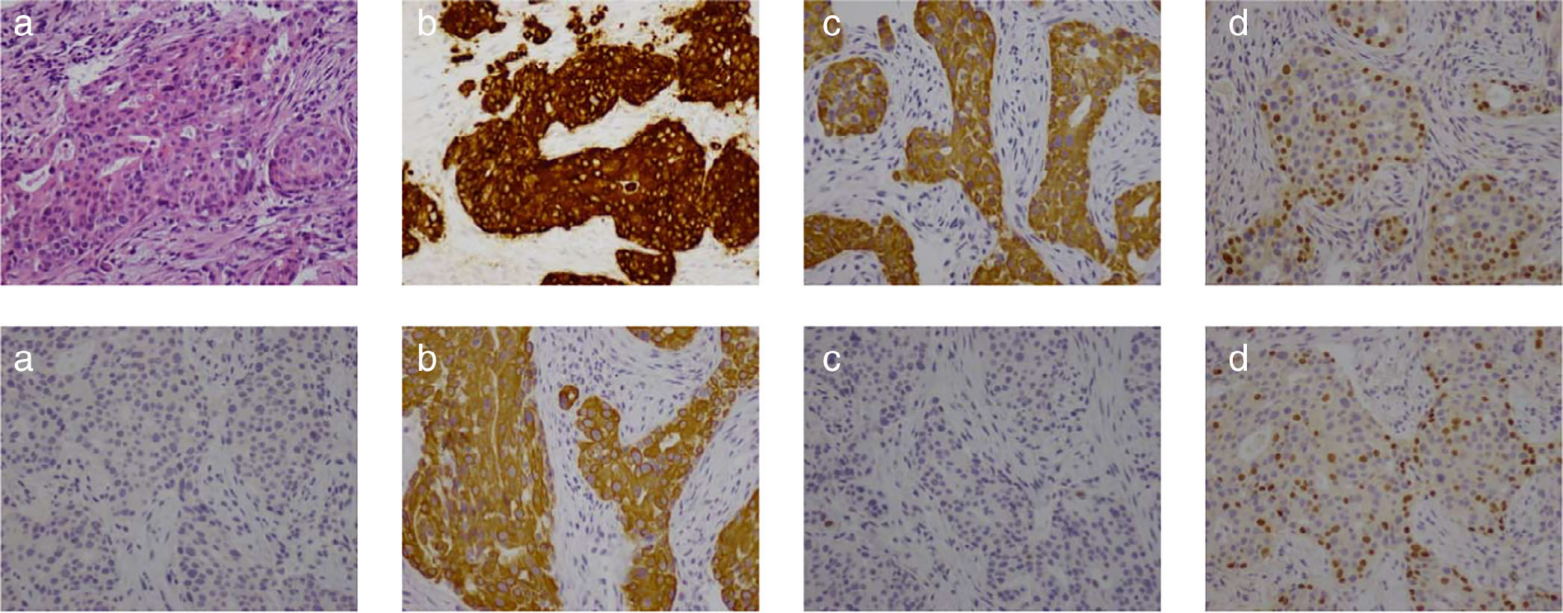
Immunobiological characteristics of case 1, Immunohistochemical analysis of lung cancer tissue. (a) Hematoxylin and eosin staining (200×); (b) ALK Ventana (DF53, 200×), (c) positive staining for CK56 (200×), (d) positive staining for P40(200×), (e) negative staining for TTF‐1 (200×), (f) positive staining for CK7(200×), (g) Naspin‐A (200×), (h) positive staining for P63 (200×)

A 54‐year‐old woman with no previous history of smoking was clinically diagnosed with NSCLC (cT2N2M1b, stage IV). A histopathological analysis of biopsied tumor tissue revealed SCC. IHC confirmed positivity for p40 and p63, negativity for TTF‐1 and napsin‐A (FIGURE 1); ALK protein was overexpressed which was confirmed via IHC ALK rearrangement. The patient received first‐line chemotherapy with four cycles of a combination of gemcitabine and carboplatin which did not suppress rapid tumor growth. The chemotherapy regimen was interrupted and replaced; the patient accepted crizotinib as second‐line treatment and showed SD but relapsed after six months.

#### Case 2: An ALK inhibitor nonresponder

**FIGURE 2:**
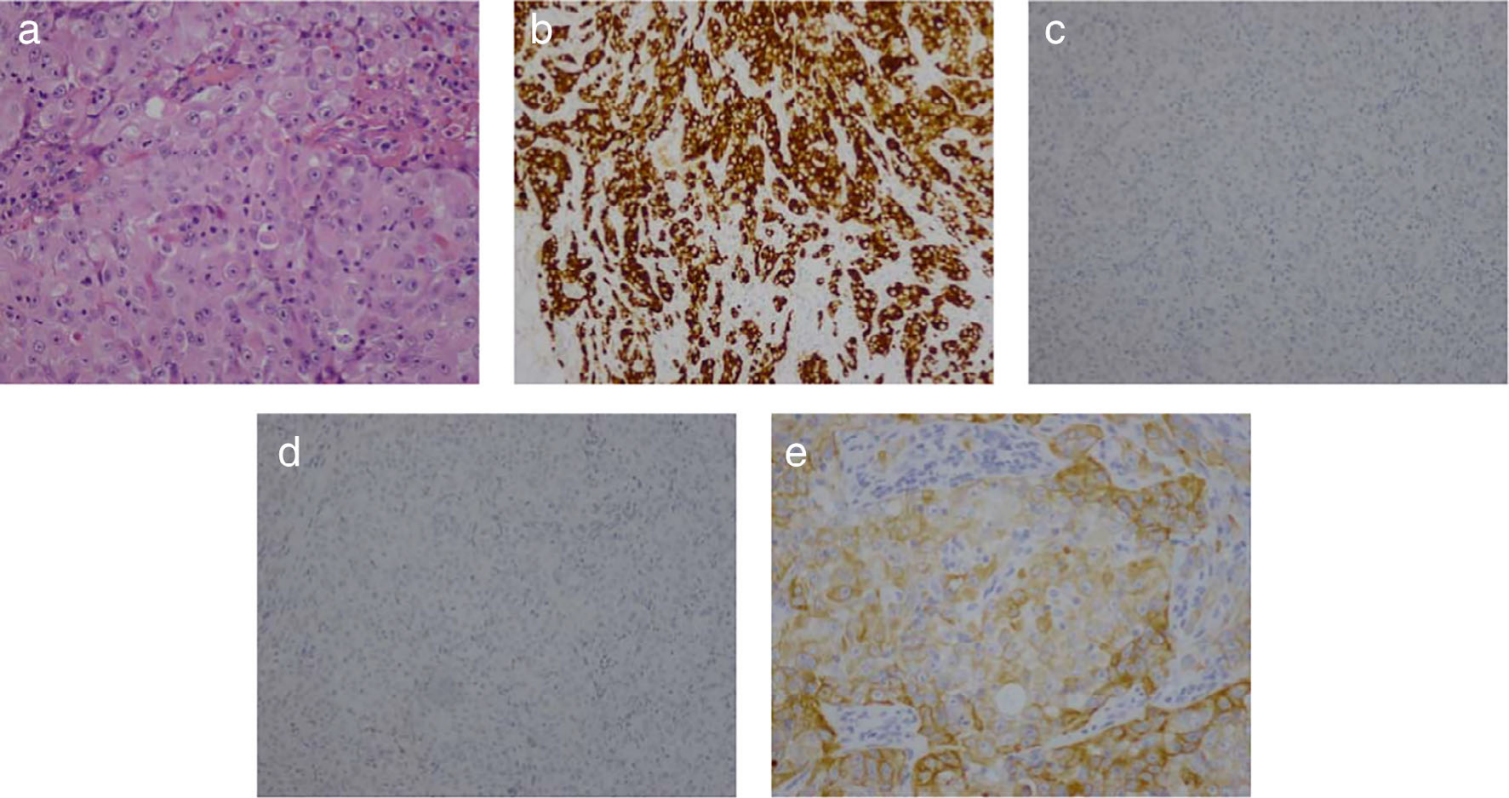
Immunohistological characteristics of case 2, Immunohistochemical analysis of lung cancer tissue. (a) Hematoxylin and eosin staining (200×); (b) ALK ventana (DF53, 200×), (c) negative staining for TTF‐1 (200×), (d) Naspin‐A (200×); (e) positive staining for CK56 (200×)

Case 2 was a 35‐year‐old woman who was a non‐smoker. She was clinically diagnosed with NSCLC (cT4N3M1b, stage IV). IHC analysis of biopsied tumor tissue revealed positive CK7 and negative TTF‐1 and napsin‐A immunoreactivity, confirming a diagnosis of SCC (FIGURE 2). Carboplatin plus taxol was administered as first‐line chemotherapy. However, this regimen was discontinued following disease progression. Rebiopsy was performed and confirmed a diagnosis of SCC and IHC ALK rearrangement. The patient accepted crizotinib as second‐line treatment but there was no response and she subsequently died of disease after one month.

#### Descriptive analysis and qualitative synthesis

We identified 31 cases of ALK rearrangement in SCC patients from a literature search (TABLE 2). In this study, a total of 31 cases were included which includes the eight patients. There was a prevalence of female (15 females, 16 male), and never smoker patients (18 never smokers, 12 smokers). The average age of patients was 55.6 ± 13.3. The mean age of female patients was 49.6, younger than 61.1 of male. The pathological staging was I + II, III and IV in three (9.6%), 10 (32.3%; IIIA eight, IIIB two) and 18 (58.1%) patients, respectively. There were eight surgically resected samples and 31 biopsies. A total of 13 (41.9%) cases were recorded as focal positive and 18 (58.1%) cases with advanced disease. In 31 cases of SCC, 10 were identified as ALK‐positive only by Ventana IHC (D5F3), one case was by NGS, 20 cases were diagnosed as ALK‐positive by Ventana IHC (D5F3) and FISH, two cases were IHC (−)/FISH (+), two cases were IHC (+)/FISH (−), and 16 cases were IHC (+)/FISH (+).

**TABLE 2:** Literature review of all clinical cases to date

Author	Age (years)	Sex	Method of diagnosis and/or type of tissue sampled	ALK detection	Stage	Smoking history (pack‐year)	Prior treatment	ALK inhibitor	Efficacy	Duration of treatment (months)
1. Kimh *et al*. 2013^10^	36	F	Bronchial biopsy of the cervical lymph node	IHC+, FISH+	IV	Non‐smoker	None	None		
2. Ochi *et al*. 2013^11^	45	F	Bronchial biopsy of cervical lymph node	IHC −, FISH +	IV	Smoker	None	None		
3. Alrifai *et al*. 2013^12^	69	M	Bronchial biopsy of cervical lymph node	IHC+, FISH+	IIIa	Smoker	Radiation	None	None	
4. Wang *et al*. 2014^13^	55	F	Bronchial biopsy of primary lesion	IHC+, FISH+	IV	Non‐smoker	PDC	Crizotinib	PR	5.8
5. Mikes *et al*. 2015^14^	36	M	Bronchial biopsy of primary lesion	IHC+, FISH+, RT‐PCR	IV	Non‐smoker	None	Crizotinib	PR	3.0 (PR maintain
6. Zhang *et al*. 2015^15^	55	F	Bronchial biopsy of primary lesion	IHC+	IV	Non‐smoker	PDC	Crizotinib	PR	6
7. Takanashi *et al*. 2015^16^	60	M	Bronchial biopsy of primary lesion	IHC+, FISH+, RT‐PCR+	II	Smoker	Adjuvant	None		
8. Vergne *et al*. 2016^17^	58	F	Bronchial biopsy of primary lesion	IHC+, FISH+	IV	Non‐smoker	None	Crizotinib	PR	7.1
9. Tamiya *et al*. 2015^18^	78	M	Bronchial biopsy of primary lesion	IHC+, FISH+	IV	Smoker	None	Alectinib	PD	1.5
10. Wang *et al*. 2016^19^	37	F	Bronchial biopsy of primary lesion	IHC+	IIIb	Non‐smoker	Radiation, PDC	Crizotinib	PR	9
11. Yamamoto *et al*. 2016^20^	76	M	Bronchial biopsy of primary lesion	IHC−, FISH+	IIIa	Non‐smoker	Radiation	None		
12. Mamesaya *et al*. 2017^21^	52	F	Bronchial biopsy of primary lesion	IHC+, FISH+	IV	Non‐smoker	PDC	Alectinib	PR	11
13. Bolzacchini *et al*. 2017^22^	51	M	Biopsy of primary lesion, surgery	IHC+, FISH+	IIIa	Non‐smoker	PDC,	Crizotinib, Alectinib	PR	14
14. Li *et al*. 2017^23^	45	F	Biopsy of primary lesion	IHC+, FISH+, NGS	IV	Non‐smoker	None	Crizotinib,	PR	9
15. Sagawa *et al*. 2018^24^	73	M	Bronchial biopsy	IHC+, FISH+	IV	Non‐smoker	None	Alectinib	PR	>9
16. Huang *et al*. 2018^25^	50	F	Lung biopsy	NGS+	IV	Smoker	PDC	Crizotinib, Alectinib	PR/PR (QTprolong)	>4
17. Watanabe *et al*. 2018^26^ Case 1	65	F	Bronchial biopsy	IHC−; FIFH+	IV	Smoker	PDC	Crizotinib, Alectinib	PD	2/1
Case 2	36	M	Bronchial biopsy	IHC+, FISH+	IIIb	Smoker	PDC	Crizotinib, Alectinib	PR	12/5
Case 3	62	M	Bronchial biopsy	IHC−, FISH+	IV	Smoker	PDC	Crizotinib	PD	1
18. Wang *et al*. 2019^27^ Case 1	63	M	Bronchial biopsy	IHC+, FISH−, NGS+	IV	Smoker	None	Crizotinib,	SD	3
Case 2	32	F	Bronchial biopsy	IHC+, FISH−, NGS+	IV	Non‐smoker	None	Crizotinib	PD	4
Case 3	53	F	Bronchial biopsy surgery	IHC+, FISH+, NGS+	IIIA	Non‐smoker	PDC	Crizotinib	SD	9
Case 4	73	F	Bronchial biopsy	IHC+, FISH+, NGS+	IV	Smoker	PDC	Crizotinib	SD	10

Abbreviations: F, female; FISH, fluorescence in situ hybridization; M, male; PD, progressive disease; PDC, platinum‐doublet chemotherapy; PR, partial response; RT‐PCR, reverse transcription polymerase chain reaction; SD, stable disease.

Eight patients underwent tumor resection. Five received postoperative adjunctive chemotherapy and attended regular check‐ups for postoperative recurrence. After regular follow‐up appointments, two patients were found to have recurrent disease. One accepted crizotinib, resulting in SD of nine months duration, the other patient accepted alectinib resulting in PR for 14 months.^22^, ^27^

Three patients^12^, ^19^, ^20^ diagnosed with stage III SCC underwent radiation therapy. After regular check‐ups, one patient was confirmed to have recurrent disease, and underwent treatment with crizotinib. This patient was considered to have obtained a PR with nine months.

This report includes all patients who were treated with at least one type of ALK‐TKI therapy and some patients had more than one treatment with different kinds of TKI. All the treatments administered for each lines of therapy were analyzed. A total of 20 patients received at least one ALK‐TKI (20 cases evaluable lines of treatment). There were 13 patients with ALK‐rearranged SCC who had previously undergone standard chemotherapy for SCC prior to treatment with crizotinib and switched regimens when ALK rearrangement was detected, when they had been diagnosed with progressive disease (PD) during chemotherapy (three cases received more than one kind of ALK‐TKI). Seven patients were treated as first‐line administration (one case was PR for three months without further information) (Table 3).

**TABLE 3:** Qualitative synthesis of reported best response

	Npts	NPR	NSD	NPD	ORR (CR + PR%)	DCR (CR + PR + SD%)	Duration time of treatment
							Mean (month)
Total patients	20	11	4	5	55.0%	75.0%	6.4 ± 4.4
First‐line treatment	7	4	1	2	57.1%	71.4%	4.5 ± 2.9
Second‐line treatment	13	7	3	3	53.8%	76.9%	7.4 ± 4.8
Smoker	7	2	2	3	28,6%	57.6%	5.6 ± 3.2
Non‐smoker	13	9	3	1	69.2%	92.3%	6.8 ± 3.6
IHC +/ FISH +	11	8	2	1	72.3%	90.1%	8.8 ± 4.4
IHC or FISH +	4	0	1	3	0	25.0%	2.8 ± 1.2

Abbreviations: DCR, disease control rate; N, member; ORR, objective response rate; PD, progressive disease; PR, partial response; SD, stable disease.

Seven patients who smoked accepted ALK treatment (two received alectinib, five received crizotinib). A total of 13 non‐smokers accepted ALK treatment (four received alectinib and nine received crizotinib). The objective response rate (ORR) was 28.6% (2/7) and 69.2% (9/13), respectively (*p* = 0.16). The disease control rate (DOC) was 57.6% and 69.2% (9/13), respectively (*p* = 0.28). The duration of treatment was almost 1.2 months longer than in smokers (smokers 5.6 ± 2.2 vs. 6.8 ± 3.6 months; *p* = 0.752). Therefore, smokers with ALK SCC who responded to crizotinib/alectinib showed a shorter duration time of treatment and worse response than non‐smokers.

In 20 cases confirmed to be ALK‐positive by IHC and FISH, 15 cases received an AIK‐inhibitor. There was one SD case in four patients assayed by IHC (+)/FISH (−) or IHC (−)/FISH (+). There were eight PR cases, two SD cases, and one PD case, out of a total of 11 patients, both IHC (+)/FISH (+). The DCR was 90.1% (10/11) and 25.0% (1/4), respectively (*p* = 0.01). Four patients in which ALK rearrangement was assayed by IHC (−)/FISH (+) or IHC (+)/FISH (−) did not respond to crizotinib, and the duration time of treatment decreased sharply from one to four months (*p* = 0.001).^26^, ^27^

## DISCUSSION

The EML4‐ALK fusion gene is one of the most important molecular alterations in NSCLC, but is extremely rarely expressed in SCC.^1^, ^2^ In our center, IHC analysis includes p40, P63, cytokeratin (CK) 5/6 and CK7, thyroid transcription factor‐1 (TTF‐1) and napsin‐A. To confirm the diagnosis of SCC requires TTF‐1 and napsin‐A completely negative and other indicators positive. In our report IHC findings were strongly suggestive that the tumors were SCC.

Caliò *et al*. reported that ALK rearrangements may be found in pure lung squamous cell carcinomas and the frequency was 2.5% (one of 40 cases) in biopsy samples. The ALK‐positive rate depends on tissue size and has been found to be higher in surgical specimens in comparison to smaller biopsy specimens.^28^, ^29^ It has been confirmed that the whole specimen obtained surgically showed typical morphohistology of SCC, and contained no other histological type. Furthermore, heterogeneous expression of ALK‐protein was seen throughout the entire area of the tumor. There was a possibility that the tumor was not pure SCC. Occasionally, the histological type of the small amount of specimen obtained via TBB was difficult to determine in subsequent IHC and molecular analyses.

In our report, five patients were surgically diagnosed with eight diagnosed via small biopsy specimens. The majority of the 31 cases in our study were diagnosed using small biopsy specimens. Fusion genes are often seen in a younger age group (mean age: 55.6 years old) and female group (15/31), especially non‐smokers.

The recommended methods for detection of ALK rearrangement include Ventana IHC (D5F3), in‐situ hybridization (FISH) and reverse transcription polymerase chain reaction (RT‐PCR).^8^, ^9^ Previous studies have found that ALK‐positive with SCC is 1%, as detected by IHC/FISH or PCR.^27^, ^30^ Because of the low frequency in squamous lung cancer, ALK fusion gene testing is not routinely recommended in the National Comprehensive Cancer Network guideline for the treatment of NSCLC. The China Food and Drug Administration (CFDA) granted full approval for crizotinib in the treatment of patients with ALK‐positive NSCLC on January 22, 2013 and Ventana IHC (D5F3) has been approved by the CFDA as an aid in identifying patients who are eligible for treatment with crizotinib.^31^ Until now, the updated CAP/IASLC/AMP molecular testing guideline for lung cancer recommended IHC as an equivalent alternative to FISH for ALK testing. Since Ventana IHC (D5F3) is the most convenient and inexpensive method for ALK rearrangement, therapeutic decisions based on IHC results are clearly reasonable.^32^

ALK‐inhibitor (crizotinib/alectinib) is much more effective than chemotherapy for ADC with harbored the ALK rearrangement. In advanced NSCLC, the disease control rate is 60% and median progression‐free survival of response is 4–10 months for first‐line combination chemotherapy. The disease control rate (DCR) decreases 20%–40% for second‐line chemotherapy and median duration of response is poor.^3^, ^30^ Morodomi *et al*. reported that NSCLC patients with EML4‐ALK fusion genes are relatively insensitive to cytotoxic chemotherapy for postoperative recurrent disease or advanced disease.^33^

Our article retrospectively analyzed the clinical features of 31 patients with ALK‐rearranged SCC. A total of 20 cases received ALK‐TKI as first‐ or second‐line treatment. The response of ALK‐TKI treatment was much better than chemotherapy. The superiority of second‐line treatment ALK‐inhibitor compared to standard chemotherapy has previously been demonstrated. The median duration time of therapy was 6.4 ± 4.4 months, much longer than patients with chemotherapy.

The therapeutic effect of ALK‐inhibitor in patients with SCC remains controversial. Target therapy with ALK‐rearranged ADC was more effective than SCC. The duration time of treatment (our study: 6.4 ± 4.4 months) ALK‐rearranged SCC was shorter than those in previous clinical trials of ALK‐rearranged ADC studies (PROFILE1007:7.7 months, PROFILE1014:10.9 months; ALESIANCT02838420:14.6 months), ^34^, ^35^ suggesting that outcomes are worse for ACC as compared to the ADC patient when ALK inhibitors are used. Therefore, ALK‐rearranged SCC patients obtained clinical benefit from ALK‐inhibitor drug treatment, but it was not as beneficial to those patients who had ALK‐rearranged ADC.

Smoking status was a negative indicator of the efficacy of ALK‐inhibitor targeted therapy for ALK‐rearranged SCC. In this study, 13 non‐smokers accepted ALK treatment and only one suffered PD, and the ORR and DCR was much higher than in those patients who were smokers. The treatment duration time was almost two months longer than those in smokers, suggesting that outcomes are worse for smokers. From these cases the implication is that non‐smoking status may be a predictive factor for treatment in ALK‐positive patients with SCC.

ALK‐positive patients with SCC detected by IHC (+)/FISH (+) tended to be positively correlated with duration of treatment and associated with initial ALK inhibitor response. Response rate and disease control rate were extremely good. The treatment duration time was almost six months longer than that of patients detected by IHC or FISH positive with ALK‐rearrangement (9.0 ± 4.2 vs. 2.8 ± 1.2 months, respectively; *p* = 0.001), suggesting better outcomes for the former as compared to the latter patient group when ALK inhibitors are used. Shi *et al*. also reported FISH (+)/IHC (−) of lung adenocarcinoma was resistant to crizotinib.^36^ It is difficult to further interpret the molecular mechanism of the lower response. The rate of discordance between ALK rearrangement detection by FISH and IHC ranges from 0.3% to 2%. So far, false‐positive explanation of IHC and FISH may be the main reason, especially with results close to the cutoff value.^37^, ^38^ Therefore, in order to devise a treatment strategy, it is reasonable to choose various methods to identify ALK‐positive patients.

Immune checkpoint blockade therapy is one of the most successful in patients with advanced NSCLC without driver oncogenes. Encouragingly, the therapeutic efficacy of immune checkpoint blockade has significantly improved for patients with advanced ACC. However, there is less data on the immune treatment of patients with ACC ALK‐rearrangement. We found only one previous case reported in the literature.^27^ With IHC, this case showed focal positivity, FISH confirmed that it was rearrangement‐negative, and next‐generation sequencing (NGS) confirmed an *ALK* point mutation (K1525E). The patient showed no response to crizotinib and remained in SD for three months following previous treatment, then subsequently received therapy with PD‐L1 immune checkpoint inhibitors. The patient's treatment continued to the last recorded visit. We seldom treat a patient in this situation, but a personalized medicine or treatment strategy is sometimes necessary. However, further case reports are required to confirm such an association.

In conclusion, because of the increasing number of reported cases of ALK SCC, this presents an opportunity to allow doctors to define specific guidelines. Molecular characteristics of lung cancer have underlined the pathogenic and therapeutic importance of specific genes involved in tumor growth. This report highlights the importance of searching for driver mutations in patients with SCC. ALK‐targeted therapy could be an effective treatment option. Non‐smoker status and IHC (+)/FISH (+) may be predictive factors in those patients. It is therefore strongly advised that ALK inhibitor drug treatment could be one of the preferred approaches in cases of non‐smoker patients with ALK SCC.
